# *STIL* enhances the development of lung adenocarcinoma by regulating the glycolysis pathway

**DOI:** 10.32604/or.2024.048562

**Published:** 2024-12-20

**Authors:** LEI WANG, XIANJIN XIE

**Affiliations:** 1 Department of Respiratory Medicine, Jen Ching Memorial Hospital, Suzhou, 215300, China; 2 Department of Respiratory Medicine, Shandong Provincial Third Hospital, Jinan, 250010, China

**Keywords:** SCL/TAL1 interrupting locus (*STIL*), Lung adenocarcinoma, E2 promoter binding factor 1, Glycolysis

## Abstract

**Background:**

To investigate SCL/TAL 1 interrupting locus (*STIL*)’s role and prognostic significance in lung adenocarcinoma (LUAD) progression, we examined *STIL* and E2 promoter binding factor 1 (E2F1) expression and their impacts on LUAD prognosis using Gene Expression Profiling Interactive Analysis (GEPIA).

**Methods:**

Functional assays including CCK-8, wound-healing, 5-ethynyl-2-deoxyuridine (EdU), Transwell assays, and flow cytometry, elucidated *STIL* and E2F1’s effects on cell viability, proliferation, apoptosis, and migration. Gene set enrichment analysis (GSEA) identified potential pathways, while metabolic assays assessed glucose metabolism.

**Results:**

Our findings reveal that *STIL* and E2F1 are overexpressed in LUAD, correlating with adverse outcomes. It enhances cell proliferation, migration, and invasion, and suppresses apoptosis, activating downstream of E2F1. Silencing E2F1 reversed the promotion effect of the *STIL* overexpression on cell viability and invasiveness. Importantly, *STIL* modulates glycolysis, influencing glucose consumption, lactate production, and energy balance in LUAD cells.

**Conclusion:**

Our model, incorporating *STIL*, age, and disease stage, robustly predicts patient prognosis, underscored *STIL*’s pivotal role in LUAD pathogenesis through metabolic reprogramming. This comprehensive approach not only confirms *STIL*’s prognostic value but also highlights its potential as a therapeutic target in LUAD.

## Introduction

Lung cancer encompasses various types, with non-small cell lung cancer (NSCLC) accounting for approximately 80%–85% of cases. Within NSCLC, lung adenocarcinoma (LUAD) is the most common subtype, making up about 40%–50% of NSCLC diagnoses, highlighting its significance in the overall landscape of lung cancer [1]. According to the latest data, lung cancer remains the leading cause of cancer deaths globally, with increasing incidence in China [2,3]. The diagnosis of LUAD currently necessitates a thorough integration of clinical radiography, molecular biology, and pathological data to precisely evaluate the prognosis and identify potential treatment targets [4]. Among them, molecular biological detection is important for improving both early diagnosis and prognosis [5,6]. Some genes, such as activated CDC42-related kinase 1 (ACK1), MET, and the stimulator of interferon genes (STING) are beneficial in predicting the prognosis of lung cancer and provide effective methods for early diagnosis [7–9]. Expanding the exploration of other biomarkers will offer greater potential for discovering innovative diagnostic techniques for LUAD.

The SCL/TAL1 interrupting locus (*STIL*) is a gene involved in regulating the cell cycle and facilitating the duplication of centrioles in dividing cells. Its function primarily involved in centrosome formation and regulating the number of spindles for chromosome segregation [10]. Genetic instability is a common feature of tumor cells. Thus, *STIL* can influence the development of chromosomal instability by controlling the structure and quantity of centrosomes, which has been linked to various malignant tumors, including prostate, colon, pancreatic, and gastric cancers [10–13]. However, the expression and prognostic significance of *STIL* in LUAD have not been reported.

Earlier studies showed that glycolysis plays an important role in the ATP synthesis in tumor cells. The Warburg effect revealed that in the tumor, cells prefer to produce lactic acid via glycolysis even when adequate oxygen is present [14–16]. The elevation of lactate levels causes an acidic environment that promotes the growth and expansion of tumor cells. Therefore, glycolysis plays an important role in the progression of tumor cells. Studies have found that oncogenes influence the progression of cancer by participating in the glycolysis process [16]. Peng et al. discovered that USP47 promoted glycolysis and NSCLC progression through BACH1 deubiquitination [17]. Fascin enhanced the glycolysis process, thus promoting both tumor growth and metastasis [18] In this study, the prognostic impact, expression, and modulation of *STIL* level in LUAD were examined and validated via immunohistochemistry and bioinformatics prediction. This study revealed that *STIL* promoted the development of LUAD by regulating the glycolysis pathway.

## Methods and Materials

### Clinical samples

A total of 10 cancer and adjacent tissue samples from LUAD patients (admitted to the hospital during March 2021 and 2022) were used in the analysis. These tissues were obtained during tumor resection surgery and obtained the samples from 7 male and 3 female patients (median age, 59 years; range, 35‒83 years). Of these, 2 had stage I + II cancers while 8 had stage III + IV. Before the study, patients were provided written consent. The study was approved by Ethics Committee of Shandong Provincial Third Hospital (DWKYLL-2021039).

### Cell culture and transfection

The human bronchial epithelial cell line (16 HBE) and lung cancer cells (NCI-H1975, H1299, H23, A549) were purchased from Procell Life Science & Technology Co., Ltd., (Wuhan, China) and grown in RPMI-1640 media (Gibco, Carlsbad, CA, USA) with 10% fetal bovine serum (FBS, Gibco, USA). Cells were maintained at 37°C with 5% CO_2_. RiboBio (Guangzhou, China) synthesized siRNA molecules targeting *STIL* (si-*STIL*-1 and si-*STIL*-2), E2 promoter binding factor 1-1 (E2F1-1), E2F1-2, and a negative control (si-NC). The amplified forms of *STIL* were inserted into the pc-DNA3.1 Vector from Tsingke Biotechnology Co., Ltd., in Beijing, China. The sequences were as follow: si-*STIL*-1: Sense, 5′ GAAUAUUUCUCAAGUUCAAGG-3′; Anti-sense, 5′ UUGAACUUGAGAAAUAUUCAG-3′; si-*STIL*-2: Sense, 5′-GAACUUUGUUCAAGAGAAAUG-3′; Anti-sense, 5′-UUUCUCUUGAACAAAGUUCUU-3′; si-E2F1-1: Sense, 5′-GAUGGUUAUGGUGAUCAAAGC-3′; Anti-sense, 5′-UUUGAUCACCAUAACCAUCUG-3′; si-E2F1-2: Sense, 5′-CACUGAAUCUGACCACCAAGC-3′; Anti-sense, 5′-UUGGUGGUCAGAUUCAGUGAG-3′; si-NC: Sense, 5′-UUCUCCGAACGUGUCACGUTT-3′; Anti-sense, 5′-ACGUGACACGUUCGGAGAATT-3′.

Logarithmic growth phase H1299 and H23 cells were plated in 6-well plates (Gibco) and grown for 24 h before transfection. The si-NC, si-*STIL*-1, si-*STIL*-2, and *STIL* overexpression vector were tansfected using Lipofectamine 2000 transfection reagent (Invitrogen, Carlsbad, CA, USA). The transfection effectiveness was determined by Western blotting. Cells in si-STIL-1 + Z-VAD-FMK group were treated with 10 µM Z-VAD-FMK (Sigma-Aldrich, St. louis, MO, USA) for 24 h after transfection.

### Western blotting

Cells were lysed with RIPA buffer (E-BC-R327, Elabscience, Wuhan, China) for protein extraction. Proteins (20 μg) were electrophoresed on SDS-PAGE and transferred to PVDF membranes which were treated with antibodies against *STIL* (ab89314, Abcam, Cambridge, UK), E-Cadherin (ab231303, Abcam), N-Cadherin (ab76011, Abcam), Vimentin (A2584, Abclonal, Wuhan, China), E2F1 (ab288369, Abcam), Hexokinase II (HK-2, ab209847, Abcam), glucose transporter 1 (Glut-1, A6982, Abclonal), Lactate dehydrogenase A (LDHA, #3582, Cell Signalling, Technology, Danvers, MA, USA), cyclin-dependent kinase 4 (CDK4, ab108357, Abcam), cyclin E1 (CCNE1, ab33911, Abcam), cyclin D1 (CCND1, ab16663, Abcam), and GAPDH (ab181603, Abcam) at 4°C overnight. It was then incubated at room temperature for 2 h with the secondary antibody (ab7090 and ab96879, Abcam). The chemiluminescent reagent Vazyme, Nanjing, China was added onto the PVDF membrane. The membrane was scanned and analyzed using the Tanon 4800 image system (Beijing Yuanpinghao Biotechnology Co., Ltd, Beijing, China), and the grayscale valure of the bands used to assess relative expression. Assessment of protein levels was performed 48 h following transfection.

### Cell counting kit-8 (CCK-8)

All respective cell lines were allowed to grow in 96-well plates (Beyotime Biotechnology, Shanghai, China), with 3 replicate wells in each group. A growth medium (100 μL/well) was placed in each well and incubated in 5% CO_2_ for 24 h at 37°C. A volume of 10 μL/well of CCK-8 detection reagent (Dojindo, Kumamoto, Japan) was then added. Theabsorbance of different time (0, 24, 48, 72, and 96 h) were detected at 450 nm via a microplate reader (BIO-TEK, Biotek Winooski, Vermont, USA).

### EdU assays

Cells were allowed to grow in 24-well plates (Thermo Fisher Scientific, Waltham, MA, USA) for 24 h. Each well was enriched with EdU medium (100 μL, 50 μmol/L, Beyotime Biotechnology) for 2 h after which cells were placed in paraformaldehyde (PFA, 4%) and 0.5% Triton X-100 solution for fixation followed by EdU staining. Nuclei were labeled with DAPI (Beyotime Biotechnology). All fluorescence microscopic images were quantified for the detection of the proportion of EdU-positive cells.

### Flow cytometry

Cells were trypsinized, rinsed with PBS, and resuspended in Binding buffer to 1 × 10^6^ cells/mL. After adding Annexin V-FITC (BD, San Jose, USA), the sample was kept for 15 min at 25°C (dark). Next, PI was added for 5 min. Results were quantified and analyzed via flow cytometry (BD) and Cell Quest software (BD).

For cell cycle detection, H1299 and H23 cells were fixed in ice-cold 70% ethanol at 4°C for 24 h, washed with PBS, incubated with a staining solution containing PI (Sigma-Aldrich, USA) and RNase A (Sigma-Aldrich) at 4°C for 30 min. Samples were loaded and assessed using a FACS Calibur flow cytometer (BD Biosciences, USA).

### Wound healing assay

H1299 and H23 cells were plated in 6-well plates and grown to monolayers after which the cell surface was scratched vertically with a sterilized tip. The detached or free-floating cells were then rinsed with PBS before the serum-free medium (SFM) was added. They were kept at 37°C with 5% CO_2_. The images were collected at 0 and 24 h.

### Transwell assay

The dissolved Matrigel gel (BD) was combined with an serum free medium and distributed uniformly across the chamber of the transwell (upper chamber surface). To turn the gel into gelatin, it was allowed to solidify at 37°C for approximately 2 h. A specific volume of the serum-free culture medium was diluted before digesting and counting the H1299 and H23 cell lines. Cells were inoculated uniformly to the upper portion of the transwell chamber, the chamber was transferred to a plate containing 10% FBS medium. The transwell cells were removed after 24 h of cell culture. Any remaining matrigel adhesive was removed using cotton swabs, and the wells were rinsed thrice with PBS. After PFA fixation and crystal violet staining, the cells migrating the the bottom of the chamber were examined by counting analysis.

### Dual-luciferase reporter assay

Initial steps involved cloning the sequence of the wild-type or mutant *STIL* promoter into the pGL3-luciferase reporter construct. After this, HEK-293T cells were co-transfected with pcDNA3.1-Vector (Vector, Tsingke Biotechnology Co., Ltd.) or E2F1 (Tsingke Biotechnology Co., Ltd.) via Lipofectamine3000 (Invitrogen) for 48 h before assessement of luciferase activity assay via a Promega luciferase activity kit.

### Gene expression analysis via RT-qPCR

Total RNA was extracted with TRIzol (Invitrogen) and reverse-transcribed to cDNA with a PrimeScript RT Reagent Kit (TaKaRa, Tokyo, Japan). qRT-PCR was conducted using a SYBR Green PCR Kit (Qiagen, Duesseldorf, Germany), on a LightCycler 480 II (Roche, Switzerland).

Beijing Tsingke Biotech supplied primers [*STIL*, F primer: 5′-CTACCCTGCAAACAGACCTCA-3′, R primer: 5′-AAAGGATATATAGGCTCCATG-3′; E2F1, F primer: 5′-GGGACTTTGCAGGCAGCGGCG-3′, R primer: 5′-GACGATCTGCGAGGAGTCGAG-3′; GAPDH (internal control), F primer: 5′-CTGCCGGTGACTAACCCTGCG-3′, R primer: 5′-GCCCAATACGACCAAATCAGA-3′]. Expression was determined via the 2^−ΔΔCt^ method.

### Chromatin immunoprecipitation (ChIP) assay

This experiment was conducted using ChIP commercial kit (cat:53008; Active Motif, Carlsbad, CA, USA). Cells were cultured into a 10 cm^2^ plate and allowed to proliferate until reaching a state of 70%–80% confluency. They were fixed and collected for further experiments. All nuclear precipitates were re-suspended in ChIP buffer for ultrasonic treatment and incubated for 24 h with a 5 μg antibody. The DNA protein complex is cross-linked and eluted through a series of washes. The DNA was prepared in TE buffer for analysis. We used RT-qPCR to evaluate *STIL*’s relative enrichment after the ChIP assay. The primer sequences were: F:5′-CGCCCCGGAACTTAACCGTTG-3′; R:Primer: 5′-TCCGCGCTCGACCAATCCCAA-3′.

### Kits analysis

Glucose uptake, lactate levels, and ATP/ADP ratios were detected by Glucose Uptake Assay Kit (J1343, Promega, Beijing, China), Lactate Assay Kit (L256, Dojindo), and ADP/ATP Ratio Assay Kit (A552, Dojindo), respectively.

### Seahorse assay

The oxygen consumption rate (OCR) and extracellular acidification rate (ECAR) were observed via the Seahorse XF96 Extracellular Flux Analyzer. (Agilent, USA), in a assay medium (Agilent, Santa Clara, CA, USA) within a non-CO_2_ incubator for 1 h under basal conditions.

### Bioinformatics analyses

The GEPIA website (http://gepia.cancer-pku.cn/index.html) was used to evaluate differences in *STIL* and E2F1 levels between normal lung tissue and LUAD tissue, and the effect of *STIL* and E2F1 on the prognosis of LUAD. Clinical data were analyzed by Cox risk regression using the survival package in R (Version 3.4.1). The relationship between *STIL* and E2F1 in The Cancer Genome Atlas Lung Adenocarcinoma (TCGA-LUAD) was tested by Spearman correlation. Kaplan-Meier curves with log-rank tests were used to assess survival, and then the expression of *STIL* and clinical indicators in TCGA-LUAD were analyzed using univariate Cox regression. LASSO regression was performed using the glmnet package method. For potential predictive factors, a Cox model was employed for estimating hazard ratios (HRs) with confidence intervals (CIs). The pROC package in R was used for drawing ROC curves. The metadata for TCGA-LUAD patients was provided in Table S1.

Gene set enrichment analysis (GSEA) was used to analyze the transcription factor for *STIL* based on the TCGA-LUAD database (ENCODE_TF_ChIP-seq_2015.txt). The JASPAR software (https://jaspar.genereg.net/) was used for predicting potential transcription factor interaction sites in the *STIL* promoter. The association between *STIL* and E2F1 was examined using R software. GSEA was also used to study the relationship between LUAD-related biological pathways and *STIL* expression based on the TCGA-LUAD database (h.all.v2023.1.Hs.entrez.gmt).

### Statistical analysis

All results were analyzed via GraphPad Prism 5.0 (GraphPad Software, La Jolla, CA, USA). The data are expressed as the mean ± standard deviation (SD). Data were compared using a two independent sample *t*-test, and the rates were evaluated via χ^2^ test or Fisher’s exact probability method. Substantial differences were indicated by *p* < 0.05. Triplicate experiments were conducted for statistical analysis.

## Results

### Overexpression of STIL in LUAD

**Figure 1:**
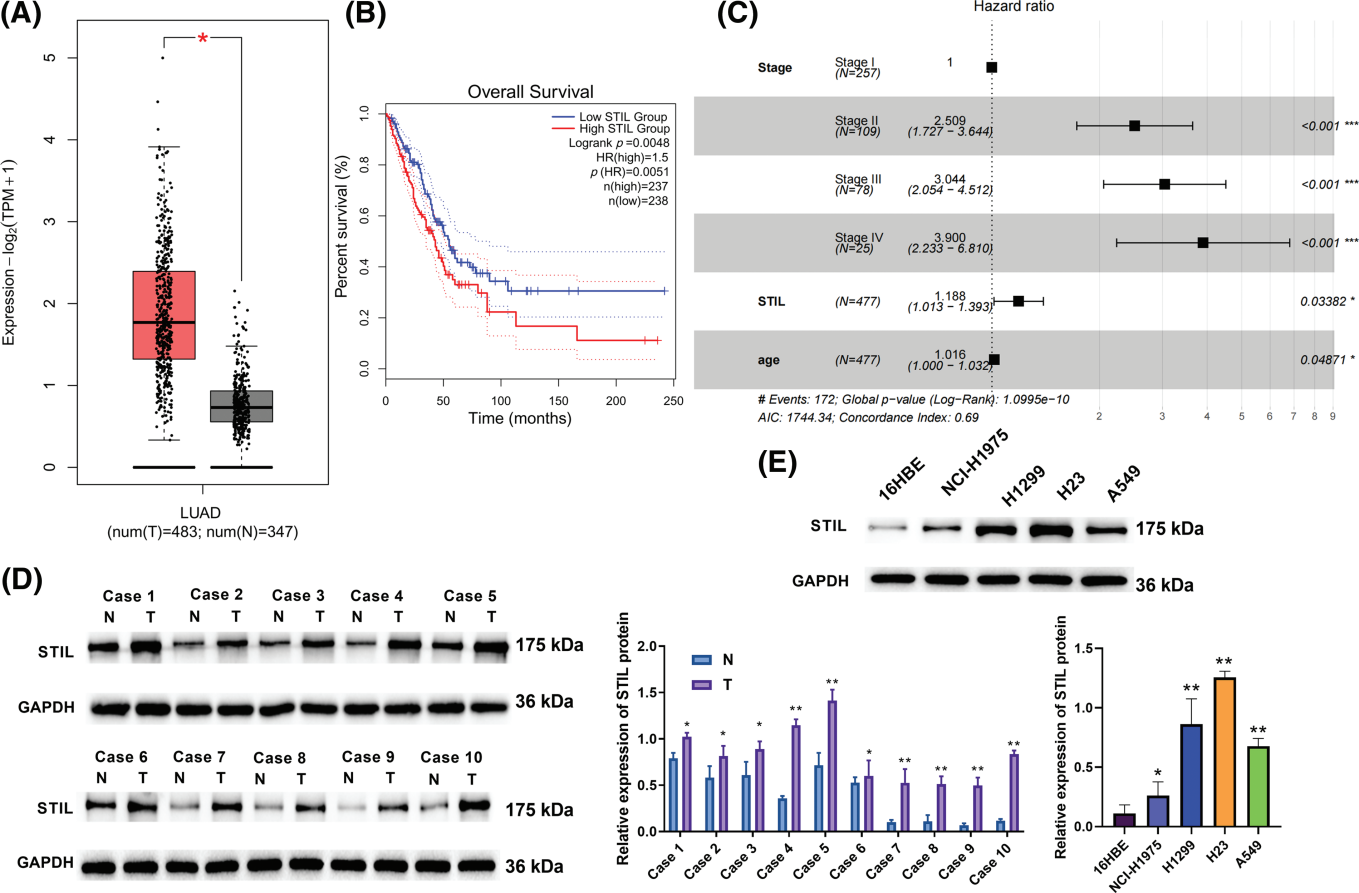
Overexpression of *STIL* in LUAD. (A) The *STIL* gene expression level was evaluated using the GEPIA database; (B) *STIL* gene association with overall LUAD patient survival; (C) Cox risk analysis of LUAD patients; (D and E) the levels of *STIL* in tissues and cells were quantified via Western blot (T: tumor, N: normal). Compared to normal or 16HBE group, *p < 0.05, **p < 0.01, ***p < 0.001.

We analyzed the expression pattern of the *STIL* gene in LUAD tissue as compared with the normal lung tissue using the GEPIA database. The results showed that *STIL* expression was markedly upregulated in LUAD tissue relative to normal lung tissue (Fig. 1A). Moreover, *STIL* levels were significantly related to the overall survival (OS). The OS rate of LUAD patients with the *STIL* low expression being substantially higher compared with those with the high *STIL* expression (Fig. 1B). Univariate and multivariate analyses were performed using the TCGA-LUAD dataset (Table 1). Through Lasso regression analysis, we selected variables combining the expression level of *STIL* with clinical indicators based on data of TCGA-LUAD. Aiming to establish a predictive model, we integrated clinical data from TCGA-LUAD patients, examining a comprehensive set of seven clinical parameters including *STIL* expression, sex, age, stage, (T_stage, N_stage, M_stage) through Lasso regression analysis (Figs. S1A, S1B). Ultimately, three variables with positive coefficients were deemed significant: *STIL* expression, age, and cancer stage (Fig. 1C). Using these, a Cox proportional hazards model was constructed with the formula: Hazard Ratio = exp (0.920 × Stage II + 1.113 × Stage III + 1.361 × Stage IV + 0.172 × *STIL* + 0.016 × age). As shown in Fig. 1C, higher stages (II, III, IV) significantly increased risk ratios (HRs) to 2.51, 3.04, and 3.90, respectively, indicating disease progression’s impact. *STIL’s* elevated levels were associated with an HR of 1.19 (*p* = 0.034), signifying its prognostic relevance. Age influenced risk marginally yet significantly (HR of 1.02, *p* = 0.049). Risk scores calculated from this model stratified patients into high and low-risk groups, revealing marked differences in survival, and underscoring the model’s predictive strength (Fig. S1C). Moreover, our model’s risk score demonstrated excellent prognostic prediction for LUAD patients (AUC = 0.683, Fig. S1D). It was also found that *STIL* was upregulated in LUAD tissue relative to normal samples (Fig. 1D). Moreover, the level of *STIL* expression in cancer cells was markedly elevated compared to normal cells (16HBE), particularly in H23 and H1299 cells (Fig. 1E). Collectively, the level of *STIL* was significantly elevated in LUAD, and increased *STIL* levels were related with unfavorable prognosis.

**Table 1:** Univariate Cox regression analysis of prognostic factors in TCGA-LUAD patients

Variable		HR (95% CI)	*p*-value
**Sex**	Female	Reference	
	Male	1.09 (0.81, 1.46)	0.588
**T_stage**	T1	Reference	
	T2	1.46 (1.02, 2.10)	0.039
	T3	3.29 (1.93, 5.59)	<0.0001
	T4	3.11 (1.63, 5.91)	0.001
**N_stage**	N0	Reference	
	N1	2.46 (1.73, 3.49)	<0.0001
	N2	3.00 (2.04, 4.41)	<0.0001
	N3	0.00 (0.00, Inf)	0.994
**M_stage**			
	M0	Reference	
	M1	2.21 (1.29, 3.79)	0.004
**Stage**	Stage I	Reference	
	Stage II	2.70 (1.87, 3.91)	<0.0001
	Stage III	3.43 (2.32, 5.07)	<0.0001
	Stage IV	3.89 (2.24, 6.77)	<0.0001
**Age**	age continuous	1.01 (1.00, 1.03)	0.027
***STIL* expression**	*STIL* continuous	1.19 (1.02, 1.38)	0.023
	*STIL*_group high	Reference	
	*STIL*_group low	0.71 (0.52, 0.95)	0.021

### Knockdown of STIL inhibited cell proliferation and promoted cell apoptosis

**Figure 2:**
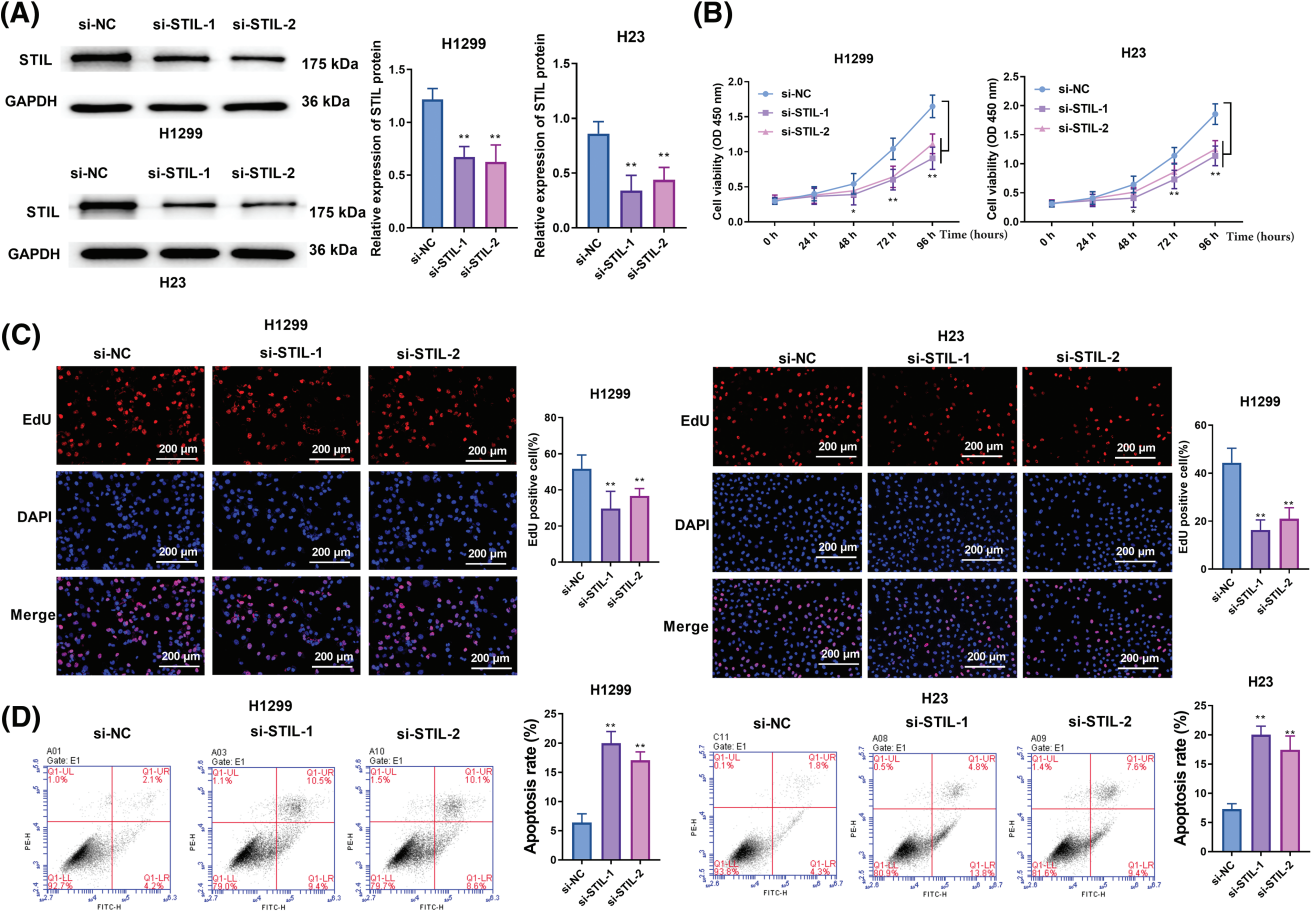
Knockdown of *STIL* suppressed cell proliferation and enhanced cell apoptosis. (A) *STIL* protein levels were determined by Western blot; cell viability, proliferation, and apoptosis were examined by CCK-8 (B), EdU (C), and flow cytometry (D), respectively. Compared to the si-NC group, *p < 0.05, **p < 0.01.

As seen in Fig. 2A and Fig. S2A, *STIL* expression was notably suppressed or elevated by transfection with siRNAs (si-*STIL*-1 and si-*STIL*-2) or *STIL*-overexpressing vectors. H23 and H1299 cell viability in si-*STIL*-1 and si-*STIL*-2 cells was substantially lower than si-NC group (Fig. 2B). Furthermore, the knockdown of *STIL* markedly reduced cell proliferation in both H23 and H1299 cell lines, as depicted in Fig. 2C. In contrast, an increase in the number of EdU upon *STIL* overexpression was observed in H23 and H1299 cells (Fig. S2B). Moreover, Knockdown of *STIL* increased cancer cell apoptosis, as shown in Fig. 2D. In contrast, *STIL* overexpression inhibited apoptosis (Fig. S2C). Collectively, *STIL* promoted LUAD cell proliferation and restrained cell apoptosis.

### Knockdown of STIL restrained cell migration, invasion, and cycle progression

**Figure 3:**
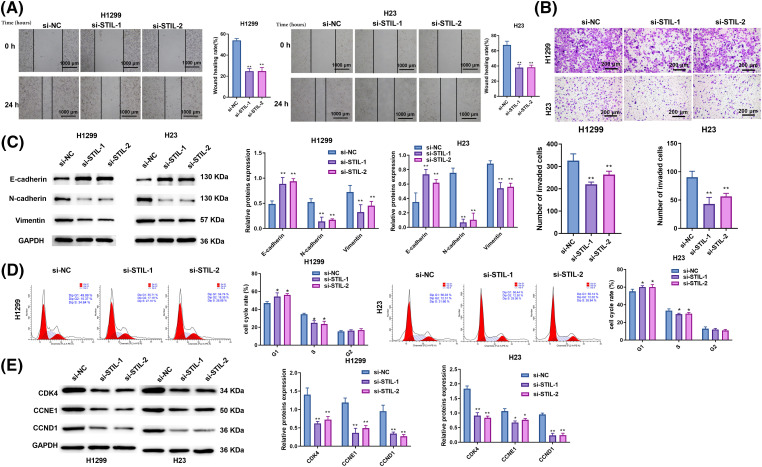
Knockdown of *STIL* restrained cell migration, invasion, and cycle progression. Invasion and migration of cells were analyzed using the wound-healing (A) and transwell (B) assays; (C) protein levels of N-Cadherin, E-Cadherin, and vimentin were measured by Western blot. (D) Cell cycle distribution was determined using flow cytometry. (E) Western blotting was used to quantify the levels of the cell cycle-associated proteins, cyclin-dependent kinase 4 (CDK4), cyclin E1 (CCNE1), and cyclin D1 (CCND1). Compared to the si-NC group, *p < 0.05, **p < 0.01.

A significant reduction in the rate of wound healing was found in both the si-*STIL*-1 and si-*STIL*-2 groups, as assessed by wound healing assays (Fig. 3A), and invading cells, as measured by Transwell assays (Fig. 3B). In contrast, *STIL* overexpression increased the wound healing rate (Fig. S2D). Moreover, the results obtained from the Western blot analysis (Fig. 3C) showed the molecular mechanisms underlying these cellular changes. Markedly increased E-Cadherin levels were observed after *STIL* knockdown, suggesting an improvement in epithelial characteristics. Simultaneously, knockdown of *STIL* induced a significant reduction in the levels of N-Cadherin and Vimentin, implying a concurrent decrease in mesenchymal features. In addition, we also found that knockdown of *STIL* caused cell cycle arrest at the G1 phase (Fig. 3D). Further measurement of the cell cycle-related proteins, CDK4, CCNE1, and CCND1, confirmed the suppression of cell cycle progression under *STIL* inhibition (Fig. 3E). Collectively, these observed alterations in protein expression substantiate the conclusion that *STIL* effectively affected the migratory, invasive, and cycle progression potential of LUAD cells.

### Up-regulation of E2F1 promoted STIL expression level

**Figure 4:**
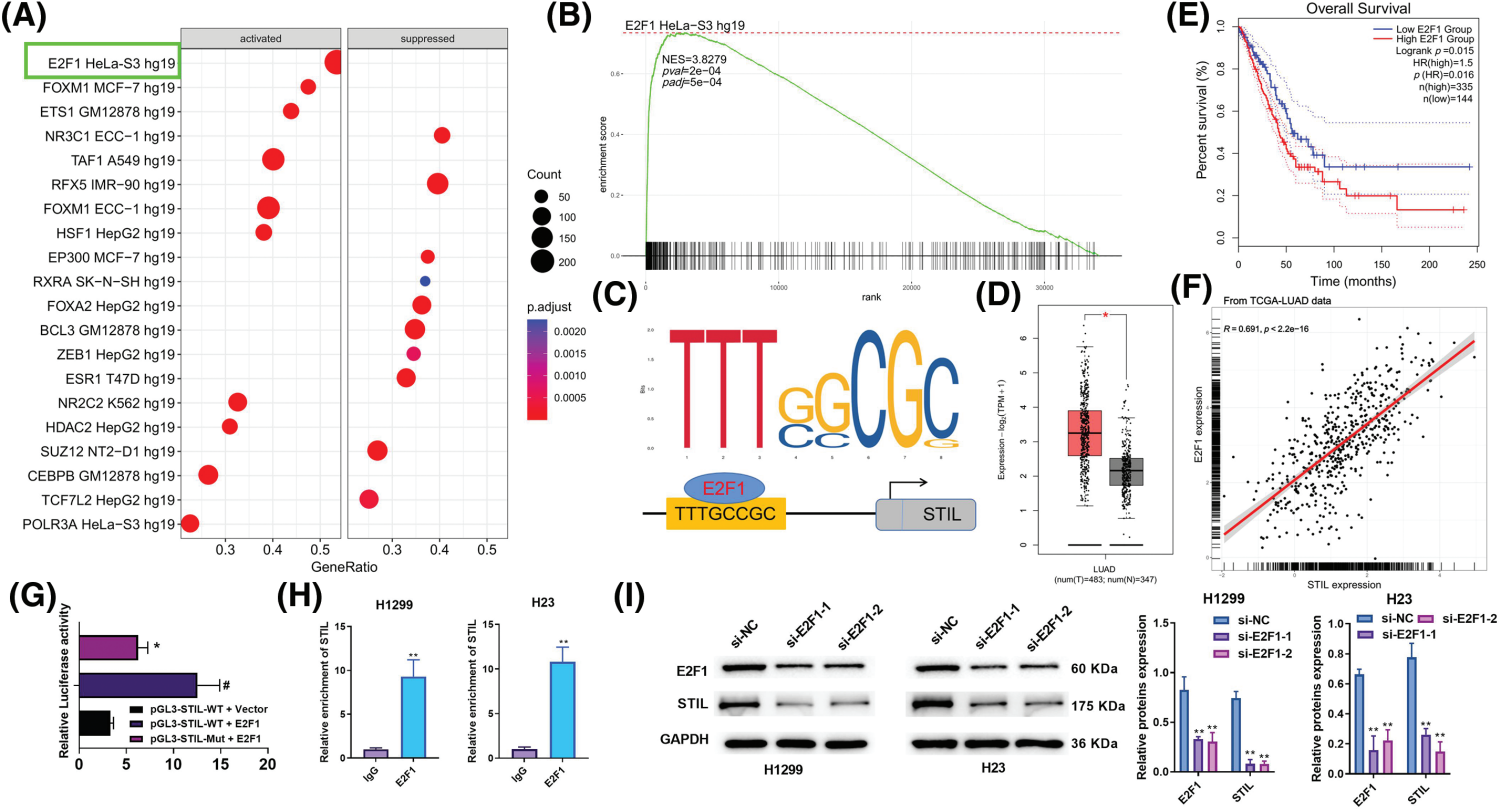
Upregulation of E2F1 promoted *STIL* expression level. (A and B) GSEA analysis of *STIL* in LUAD using data from “ENCODE_TF_ChIP-seq_2015.txt”; (C) possible transcription factor binding sites of *STIL* are predicted by the JASPAR software; (D) analysis of the E2F1 gene expression level using the GEPIA database; (E) the association between the E2F1 gene and the overall survival rates of patients with LUAD; (F) correlation analysis of E2F1 and *STIL*; dual-luciferase reporter assay (G) and ChIP assay (H) was used to verify the interaction of *STIL* and E2F1; (I) protein level of E2F1 and *STIL* were tested by Western blot. Compared to the pGL3-STIL-WT + Vector group, ^#^*p* < 0.05. Compared to pGL3-STIL-WT + E2F1 or si-NC groups, *p < 0.05, **p < 0.01.

Based on a single-gene GSEA analysis of TCGA-LUAD using data from “ENCODE_TF_ChIP-seq_2015.txt”, the upstream mechanism of *STIL* prediction was examined. The results indicate that E2F1 potentially functions as a transcription factor that positively regulates the level of *STIL* (Figs. 4A, 4B). It was noted that the *STIL* promoter contains a possible E2F1 binding site upstream of the translation initiation site using the JASPAR software to predict potential transcription factor binding sites (Fig. 4C). Further, GEPIA analysis found that E2F1 was higher expressed in LUAD patients’ samples, and higher E2F1 was correlated with poor prognosis (Figs. 4D, 4E). A positive correlation was observed between E2F1 and *STIL* expression in LUAD tissues (Fig. 4F). The *STIL*-E2F1 interaction was verified using dual-luciferase reporter experiment and ChIP assays. The results of the experiment indicated that the luciferase activity of *STIL* promoter-WT-transfected cells was markedly enhanced by E2F1 upregulation. In contrast, the stimulatory impacts of E2F1 upregulation on luciferase activity were considerably reduced when the binding sequence was mutated. This suggests that E2F1 may interact with the promoter of *STIL* to increase its expression (Fig. 4G). Relative enrichment of the *STIL* promoter in the E2F1 group was markedly greater than in the IgG group (Fig. 4H). Further, the down-regulation of E2F1 remarkably inhibited the level of *STIL* (Fig. 4I). Altogether, E2F1 promoted the *STIL* expression level. Moreover, the findings of Fig. 4I also confirmed the successful transfection of E2F1 siRNA into H1299 and H23 cells.

### Effects of E2F1 on cell viability, invasion ability, and cell cycle were regulated by STIL

**Figure 5:**
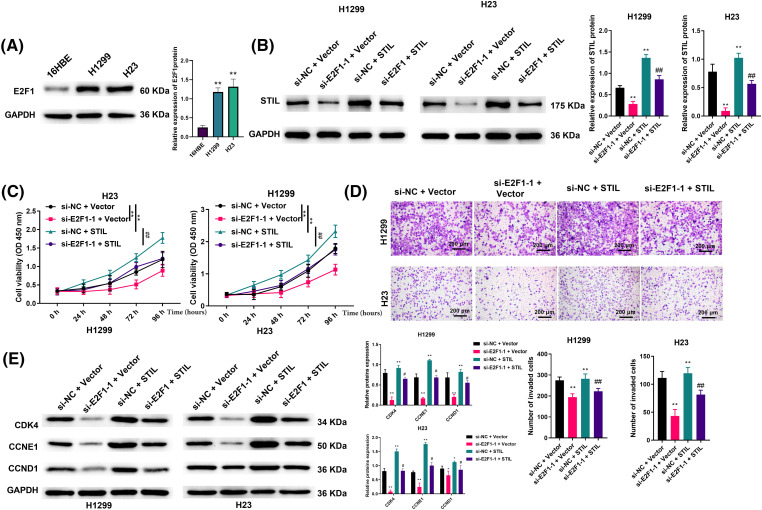
Effects of E2F1 on cell viability and invasion ability were regulated by *STIL*. (A and B) protein levels of E2F1 and *STIL* were determined by Western blotting; cell viability and invasion were evaluated by CCK-8 (C) and transwell (D) assays. (E) Western blotting was used to measure the expression levels of the cell cycle-related proteins, CDK4, CCNE1, and CCND1. Compared to the si-NC + Vector group, *p < 0.05, **p < 0.01. Compared to the si-E2F1-1 + Vector group; ^#^*p* < 0.05, ^##^*p* < 0.01.

Associations between E2F1 and *STIL* in cell viability and invasion were further studied. Firstly, we verified the expression levels of E2F1 in 16HBE, H1299, and H23. This showed that E2F1 levels were markedly higher in the LUAD cells relative to the normal cells (Fig. 5A). Secondly, we transfected *STIL* into cells. We found that after transfection, the level of intracellular *STIL* was markedly up-regulated, suggesting that the transfection was successful (Fig. 5B). As shown in Fig. 5B, down-regulation of E2F1 could significantly reduce the expression level of *STIL*. In contrast, overexpression of *STIL* could reverse this reduction. Moreover, relative to the si-NC + Vector group, the knockdown of E2F1 significantly reduced cell viability, invasion, and the levels of cycle-related proteins, while overexpression of *STIL* could significantly increase cell viability, invasion, and the levels of cycle-related proteins. Meanwhile, compared with the si-E2F1-1 + Vector group, overexpression of *STIL* counteracted the effects of E2F1 knockdown on cell viability, invasion, and the levels of cycle-related proteins (Figs. 5C–5E). Collectively, the influence of E2F1 on cell viability, invasion ability, and cell cycle was mediated by *STIL*.

### STIL regulated glycolysis

**Figure 6:**
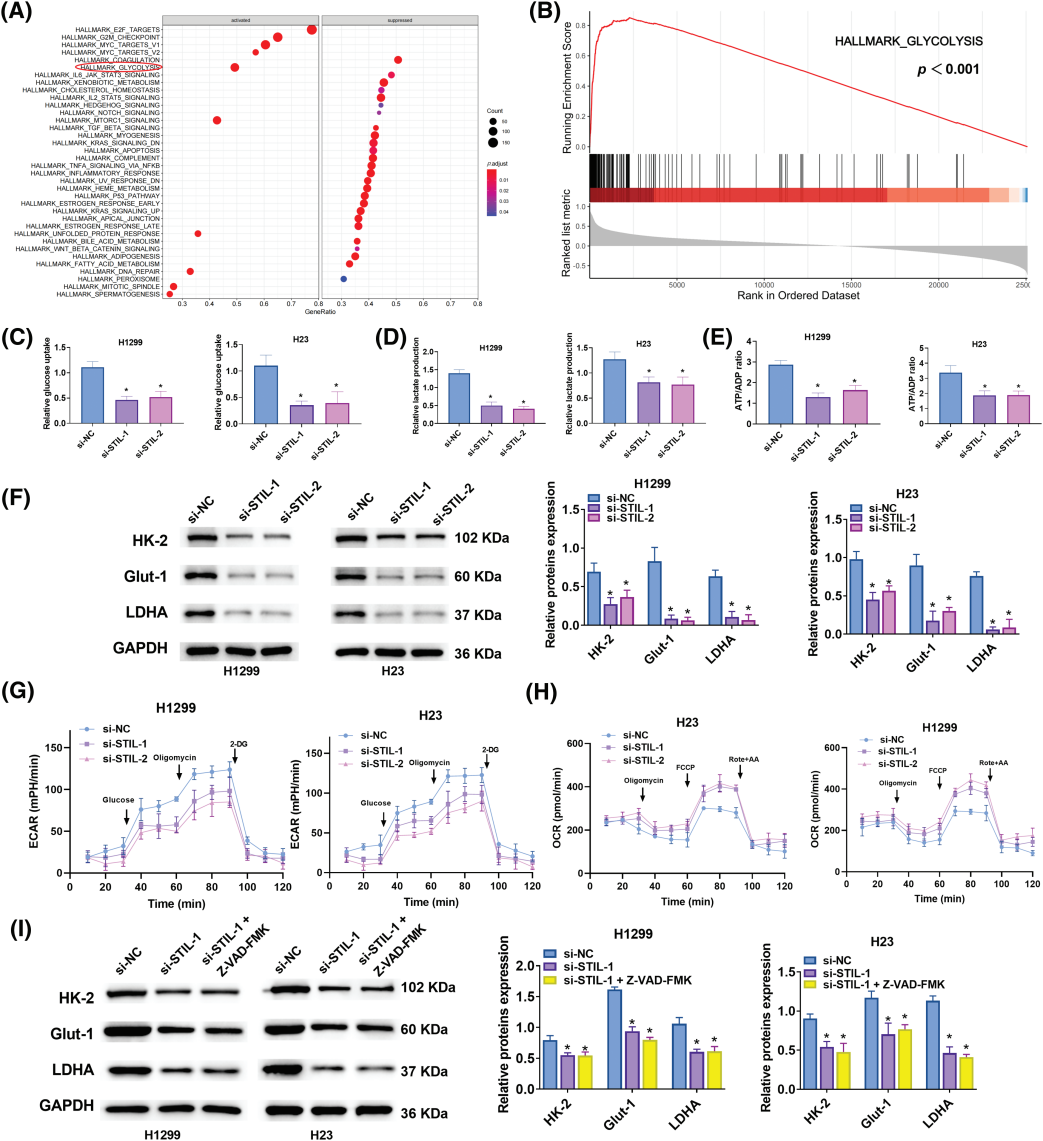
Glycolysis regulated by *STIL*. (A and B) GSEA analysis of *STIL* in LUAD using data from “h.all.v2023.1.Hs.symbols.gmt”; (C–E) Glucose absorption, lactate production, and ATP/ADP ratio were assessed using kits; (F) Protein levels of HK-2, GLUT-1, and LDHA were assessed by Western blot. (G) ECAR and (H) OCR in LUAD cells after *STIL* knockdown. (I) The protein levels of HK-2, GLUT-1, and LDHA were tested by Western blot after treatment with Z-VAD-FMK. Compared with the si-NC group; *p < 0.05.

GSEA enrichment analysis, leveraging the TCGA-LUAD database (h.all.v2023.1.Hs.entrez.gmt), identified glycolysis as a significantly associated pathway with *STIL* expression (Figs. 6A, 6B). Further investigations revealed that downregulation of *STIL* notably inhibited intracellular glucose uptake, lactate production, and the ATP/ADP ratio (Figs. 6C–6E). Conversely, overexpression of *STIL* enhanced glycolytic processes, including glucose uptake, lactate levels, and theATP/ADP ratios in H1222 and H23 cells (Figs. S2E–S2G). Additionally, *STIL* downregulation reduced the protein levels of key glycolytic enzymes HK-2, GLUT-1, and LDHA (Fig. 6F), while their levels increased following *STIL* overexpression (Fig. S2H). Notably, *STIL* knockdown significantly diminished the ECAR (Fig. 6G) and elevated the OCR (Fig. 6H), suggesting a shift from aerobic glycolysis towards enhanced aerobic oxidation. To further explore *STIL’s* regulatory role in glycolysis, we employed the apoptosis inhibitor Z-VAD-FMK. The findings indicated that *STIL* downregulation continued to decrease the protein levels of HK-2, GLUT-1, and LDHA even after treatment with Z-VAD-FMK (Fig. 6I). The results revealed that the effect of *STIL* on the expression of key glycolysis enzymes was not caused by apoptosis. Collectively, the results demonstrate the involvement of *STIL* in regulating glycolysis in LUAD.

## Discussion

Lung adenocarcinoma incidence is more than that of other subtypes, and the development and incidence of LUAD have demonstrated an upward trend recently [19,20]. Patients with early lung adenocarcinoma generally have no typical manifestations, which leads to the failure to detect lung cancer in time, delays the best time for treatment, and leads to poor prognosis [21].

Further, the incidence of lung cancer is substantially elevated as a result of the impact of multiple factors, including long-term smoking, working environment, climate change, etc. [22]. The association between CEP85 and *STIL* was identified in a study by Liu et al. This association facilitates cell migration. This discovery adds to the growing body of evidence emphasizing *STIL*’s multifaceted roles in cancer biology, particularly in the context of cell migration, a critical component of cancer metastasis [23]. Concurrently, Ouyang et al. reported *STIL* upregulation in nasopharyngeal carcinoma tissues. Further, Wang et al. discovered that *in vivo* and *in vitro*, *STIL* depletion blocked the growth and metastasis of lung cancer. In contrast, excess *STIL* promotes cancer cell invasion and migration by triggering the EMT [24]. Importantly, it was found that *STIL* not only promoted proliferation but also aided migration and invasion. These observations highlight the multifaceted roles of *STIL* in multiple types of cancer, representing that it may serve as a target for therapeutic interventions [25].

This study focused on examining the expression and prognosis of *STIL* in LUAD, as well as its influence on the progression of LUAD. The expression of *STIL* was discovered to be considerably elevated in LUAD, and this was found to be correlated with a negative prognosis. These findings suggest potential use as a biomarker and target for treatment. Meanwhile, we constructed a multifactor Cox risk regression model by combining the expression of *STIL* with clinical data. The risk score is a reliable predictor of the survival outcome for patients with LUAD. We also examined *STIL*’s role in LUAD growth and metastasis, revealing its involvement in cell propagation, dissemination, invasion, and cell death. Similar effects were observed in gastric cancer cells upon *STIL* inhibition [13]. Overall, *STIL* may function as an oncogene in LUAD and contribute to the development of tumors.

To further elucidate the mechanism underlying *STIL*’s action in LUAD, additional experiments were conducted. A study by Erez et al. revealed that the transcription factor E2F1 could interact with the *STIL* promoter. They showed that overexpression of E2F1 induced the expression of *STIL*, whereas knockdown of E2F1 led to downregulated *STIL* expression [26]. Similarly, we found that the *STIL* promoter was bound by transcription factor E2F1 and activated by E2F1. Moreover, E2F1 suppression inhibited cellular invasion and viability. However, *STIL* overexpression reversed this effect. The findings reveals a possible regulatory pathway that includes E2F1 and *STIL*, giving a better understanding of how *STIL* impacts LUAD progression.

Recently, multiple studies have emphasized the importance of active glycolysis as a key promotor in tumorigenesis [27,28]. Importantly, the function of PRDM14 in promoting chemoresistance in A549/cisplatin-resistant cells, including their progenitor A549 human lung adenocarcinoma cells, has been elucidated by He et al. [29]. Furthermore, Li et al. have revealed that heightened expression of GLUT-4 not only enhances cell propagation but also drives glycolysis, with significant effects for NSCLC patients. Collectively, these findings present convincing evidence regarding the pivotal significance of glycolysis in the advancement of lung cancer [30]. In this study, glycolysis product or key enzymes, including HK-2, GLUT-1, LDHA, and ATP/ADP ratio, was inhibited via the knockdown of STIL. Taken together, it is predicted that STIL reduction has an antitumor effect on LUAD via the inhibition of glycolysis. This finding is consistent with the findings of previous studies.

## Conclusion

In conclusion, *STIL* is remarkably highly expressed in LUAD and its elevated level can be used as a marker of unfavorable prognosis in LUAD. This gene, which is anticipated to become a novel target for the detection and intervention of LUAD patients, is activated by E2F1 and influences the progression of LUAD by modulating glycolysis. However, the precise mechanisms by which *STIL* controls lung cancer cell migration need to be further studied. The complexities of *STIL*’s function in the progression of cancer biology require further investigation.

## Supplementary Materials

Figure S1 Establishment of prognostic model. (A) Regularization path of lasso regression for clinical variables in TGCA-LUAD patients. (B) Cross-validation for optimal lambda selection in Cox regression Model. (C) Impact of risk score on LUAD patient overall survival. (D) Evaluating the predictive accuracy of the LUAD prognostic model.

Figure S2 Effects of STIL overexpression on LUAD cell proliferation, apoptosis, migration, and glycolysis. (A) STIL level in cells was measured by Western blot. Cell proliferation, apoptosis, and migration were tested by EdU (B), Flow cytometry (C), and wound healing assay (D), respectively. (E) Protein levels of HK-2, GLUT-1, and LDHA were tested by Western blot. (F-H) Glucose uptake, lactate production, and ATP/ADP ratio were tested by kits. * *p* <0.05, ** *p* <0.01, compared with the STIL group.

## Data Availability

The data that supported the findings of this study are available from the corresponding author upon reasonable request.

## Supplementary Materials

The supplementary material is available online at https://doi.org/10.32604/or.2024.048562.
