# Supplementary figures and images for: STIL enhances the development of lung adenocarcinoma by regulating the glycolysis pathway
Source: Oncol Res. 2024 Dec 20;33(1):123–32. doi: 10.32604/or.2024.048562 (PMC11671407; doi:10.32604/or.2024.048562)

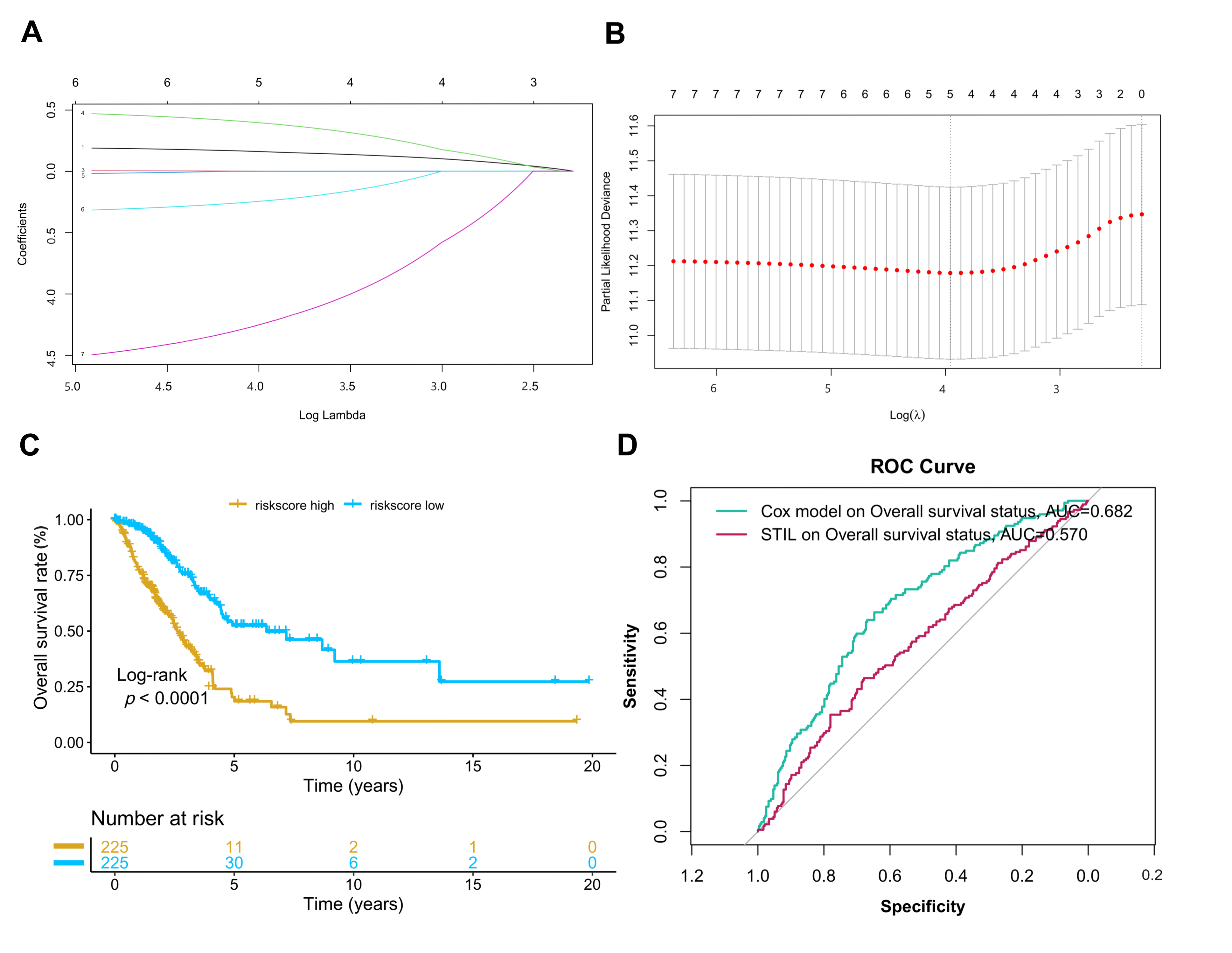

Supplement: Figure S1 — (A) Regularization path of lasso regression for clinical variables in TGCA-LUAD patients. (B) Cross-validation for optimal lambda selection in Cox regression Model. (C) Impact of risk score on LUAD patient overall survival. (D) Evaluating the predictive accuracy of the LUAD prognostic model. [file OncolRes-33-48562-s001.tif]

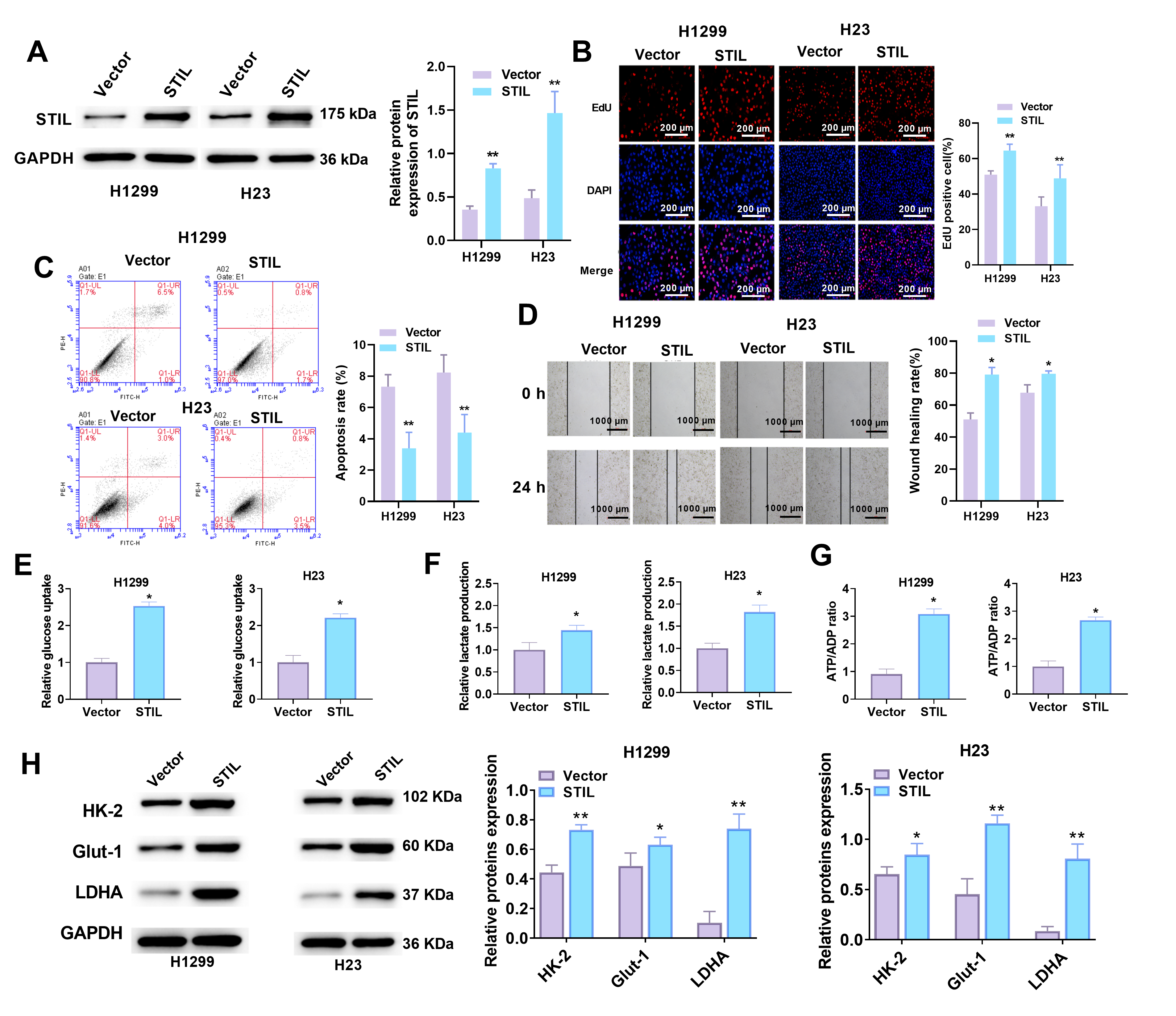

Supplement: Figure S2 — (A) STIL level in cells was measured by Western blot. Cell proliferation, apoptosis, and migration were tested by EdU (B), Flow cytometry (C), and wound healing assay (D), respectively. (E) Protein levels of HK-2, GLUT-1, and LDHA were tested by Western blot. (F-H) Glucose uptake, lactate production, and ATP/ADP ratio were tested by kits. * p <0.05, ** p <0.01, compared with the STIL group. [file OncolRes-33-48562-s002.tif]
